# Antimicrobial and Antibiofilm Activities of Probiotic Lactobacilli on Antibiotic-Resistant *Proteus mirabilis*

**DOI:** 10.3390/microorganisms8060960

**Published:** 2020-06-26

**Authors:** Mona Shaaban, Ola A. Abd El-Rahman, Bashair Al-Qaidi, Hossam M. Ashour

**Affiliations:** 1 Department of Microbiology and Immunology, Faculty of Pharmacy, Mansoura University, Mansoura 35516, Egypt; mona_ibrahem@mans.edu.eg; 2 Department of Microbiology and Immunology, Faculty of Pharmacy (Girls), Al-Azhar University, Cairo 11651, Egypt; olaali.pharmg@azhar.edu.eg; 3 Madinah Maternity and Children Hospital, Madinah 42319, Saudi Arabia; bashairzuhair20@gmail.com; 4 Department of Biological Sciences, College of Arts and Sciences, University of South Florida St. Petersburg, St. Petersburg, FL 33701, USA; 5 Department of Microbiology and Immunology, Faculty of Pharmacy, Cairo University, Cairo 11562, Egypt

**Keywords:** *Proteus mirabilis*, antibiofilm, anti-adherence, *Lactobacillus*

## Abstract

The emergence of biofilm-forming, multi-drug-resistant (MDR) *Proteus mirabilis* infections is a serious threat that necessitates non-antibiotic therapies. Antibiotic susceptibility and biofilm-forming activity of *P. mirabilis* isolates from urine samples were assessed by disc diffusion and crystal violet assays, respectively. Antimicrobial activities of probiotic *Lactobacilli were evaluated by agar diffusion. Antibiofilm and anti-adherence activities were evaluated* by crystal violet assays. While most *P. mirabilis* isolates were antibiotic-resistant to varying degrees, isolate P14 was MDR (resistant to ceftazidime, cefotaxime, amoxicillin-clavulanic acid, imipenem, ciprofloxacin, and amikacin) and formed strong biofilms. Cultures and cell-free supernatants of *Lactobacillus casei* and *Lactobacillus reuteri* exhibited antimicrobial and antibiofilm activities. The 1/16 concentration of untreated supernatants of *L. casei* and *L. reuteri* significantly reduced mature biofilm formation and adherence of P14 by 60% and 72%, respectively (for *L. casei*), and by 73% each (for *L. reuteri*). The 1/8 concentration of pH-adjusted supernatants of *L. casei* and *L. reuteri* significantly reduced mature biofilm formation and adherence of P14 by 39% and 75%, respectively (for *L. casei*), and by 73% each (for *L. reuteri*). Scanning electron microscopy (SEM) confirmed eradication of P14’s biofilm by *L. casei*. *L. casei* and *L. reuteri* could be utilized to combat *Proteus*-associated urinary tract infections.

## 1. Introduction

*Proteus mirabilis* is one of the most common pathogens associated with urinary tract infections (UTI) [1,2]. In addition, it can cause surgical wound infections, biliary tract infections, wound infections, and nosocomial infections [3]. It is part of the normal gut flora and can cause opportunistic infections in immunocompromised and elderly patients [3]. The emergence of antibiotic resistance in clinical isolates of *P. mirabilis* is a major healthcare issue [4,5]. *P. mirabilis* poses a major challenge in infection management as it produces AmpC β-lactamases and extended spectrum β-lactamases [6]. Furthermore, it can develop complex biofilms with accumulated layers of polysaccharides in which sessile cells are embedded, which adds to the severity of the infection [7]. The severity, chronicity, and dissemination of *Proteus* infections have been mainly attributed to its ability to form biofilms [7].

Probiotics are living microorganisms that belong to *Lactobacillus* and *Bifidobacterium* genera [8,9]. The intestinal probiotic *Lactobacillus* strains have been recognized for their antimicrobial activities against enteric bacterial pathogens [10]. Different groups showed that *Lactobacillus casei* produces bacteriocins and anti-adherence biosurfactant proteins against *Staphylococcus aureus*, *Bacillus subtilis*, and *Micrococcus roseus* [11,12]. Human gastrointestinal *Lactobacillus reuteri* produces a broad-spectrum antimicrobial called reuterin, which possesses activity against Gram-positive and Gram-negative enteric pathogens [13]. Probiotic *Lactobacilli* can also suppress virulence and dissemination of infectious pathogens [14]. This is typically accomplished through the production of organic acids and antimicrobials, such as bacteriocins, lipopeptides, and surface proteins [11,15,16].

We have previously shown that probiotic lactobacilli inhibited growth, biofilm formation, and gene expression of *Streptococcus mutans* [17]. The anti-infective and anti-colonization properties of probiotic lacobacilli are of paramount importance in combating various bacterial infections [18,19]. Due to their antimicrobial properties, probiotics may be considered for treatment and prevention of infectious diseases caused by oral, enteric, and urogenital pathogens [20,21]. *Lactobacilli* have been specifically shown to prevent recurrent urinary tract infections [22]. This indicates their promise as anti-*Proteus* agents. Given the antibiotic resistance problem, it is important to develop *Lactobacillus*-based approaches to combat *Proteus mirabilis*-induced urinary tract infections and catheter-associated infections. 

In order to be able to better manage *P. mirabilis* infections, we investigated the potential inhibitory activities of *L. casei* and *L. reuteri* on bacterial growth, mature biofilm formation, and adhesion properties of multi-drug-resistant (MDR) *Proetus mirabilis* clinical isolates.

We tested pH-adjusted supernatants of *Lactobacillus*, which is a commonly used approach in *Lactobacillus* probiotic studies to reduce the acidity of the *Lactobacillus* supernatants and thus assess if the antimicrobial activity of *Lactobacillus* products is pH-dependent [23,24,25,26,27]. This is significant given that studies showed that the acidic pH (due to lactic acid secretion) contributed only a small part of the activity [28], or did not contribute any additional activity (activity was pH-independent) [29]. In our study, the pH adjustment was important to reveal how much of the anti-*Proteus* activity of probiotic *Lactobacillus* supernatants was related to non-acidic products and whether the antimicrobial activity of the supernatant was pH-dependent. 

## 2. Materials and Methods

### 2.1. Microorganism and Growth Conditions

*P. mirabilis* isolates were obtained from urine samples collected from several hospitals in Madinah, KSA (Ohud Hospital, King Fahad Hospital, Al-Ansar Hospital, and Madina Maternity and Children Hospital). *P. mirabilis* isolates were identified using biochemical assays and confirmed as *P. mirabilis* using the VITEK compact system. Assays included Gram stain, growth characteristics on MacConkey agar, CLED agar, and triple sugar iron media [30]. 

Two probiotic strains (*L. casei* DSM 20011 and *L. reuteri* DSM 20016) were purchased from MERCEN, Egypt. *Lactobacillus* isolates were grown on deMan, Rogosa and Sharpe medium (MRS) and were anaerobically (AnaeroGen 2.5 L sachets in 2.5 L AnaeroJar AG25, Oxoid Ltd., Hampshire, UK) incubated at 37 °C [31].

Ethical approvals were obtained from the Institutional Review Boards of the hospitals, ethics committees of the college of pharmacy, Taibah University, KSA and Faculty of Pharmacy, Mansoura University, Egypt. Ethical approval code is TUCODRE/20151025/ALQAIDI (in October 2015). 

### 2.2. Antimicrobial Susceptibility of P. mirabilis Isolates

*P. mirabilis* isolates were tested for their resistance to different antimicrobials using the disk diffusion method [32]. The antimicrobials used were amoxicillin-clavulanic acid (AMC; 30 mg), imipenem (IMP; 10 mg), cefoxitin (FOX; 30 mg), ceftazidime (CAZ; 30 mg), ciprofloxacin (CIP; 5 mg), cefotaxime; (CTX; 30 mg), and amikacin (AK; 30 mg) (Bioanalyse, Ankara, Turkey).

### 2.3. Detection of Biofilm Formation by P. mirabilis Isolates

The mature biofilm of *P. mirabilis* isolates was formed in flat-bottomed 96-well microtiter plates. The overnight culture of each isolate was adjusted to 0.5 McFarland (1.5 × 10^8^ CFU/mL) using Muller Hinton broth. Then, 200 µL of the diluted cultures was distributed in each well and incubated at 37 °C for 48 h. The planktonic cells were removed and the attached cells were gently washed twice with sterile physiological saline. Then, 200 µL of methanol (99%) was added to each well and retained for 15 min to fix the sessile cells. The methanol was discarded, and the plate was left until complete dryness. To stain the adherent cells, 200 µL of 2% *w*/*v* crystal violet solution was added to each well and was left for 20 min. The wells were washed gently and left to dry. Glacial acetic acid (200 µL; 33% *w*/*v*) was added to release the bound dye and the absorbance was measured at OD_540_ nm using ELISA microplate reader (MR-960, Perlong Medical Equipment Co. Ltd., Nanjing, China). Depending on the optical density (OD) generated by the bacterial biofilms, the tested *P. mirabilis* isolates were classified into non-biofilm producers, weak biofilm producers, moderate biofilm producers, and strong biofilm producers [33]. The cut-off OD of the negative control (ODc) was determined by adding the mean of the negative control to three standard deviations of it. The lack of biofilm formation is indicated by ODs ≤ ODc for the tested isolates. Weak biofilm production is indicated by ODc < OD ≤ (2 ODc). Moderate biofilm formation is indicated by (2 ODc) < O.D. ≤ (4 ODc). Strong biofilm formation is indicated by (4 ODc) < O.D. Tests were conducted in triplicates. 

### 2.4. Preparation of Cell-Free Supernatant from Lactobacillus Strains

In order to prepare the *Lactobacillus* culture, the MRS medium was inoculated with *Lactobacillus* strains with inoculum size 1% *v*/*v* and was incubated anaerobically at 37 °C for 48 h. The grown culture was centrifuged at 6000 rpm for 15 min to separate all cells and the supernatant was filtered through a 0.22 µm membrane filter. The cell-free supernatant was labeled as untreated supernatant (U) and stored at −20 °C. The *Lactobacillus* supernatant was highly acidic due to lactic acid production. To adjust the pH of the supernatant to pH 6.5–7.0, 1N NaOH was used and this fraction of the supernatant was labeled as treated supernatant (T) and stored at −20 °C [34]. 

### 2.5. Antimicrobial Activity of Lactobacillus Supernatants

The antimicrobial activity of *Lactobacillus* supernatants (treated or untreated) was assessed against the *P. mirabilis* isolates P2, P4, P14, P15, P23, P24 and P25 using microtiter plate assays. The antibacterial activity was assayed by the agar diffusion method [35]. The *P. mirabilis* isolates were incubated in Muller Hinton broth at 37 °C for 24 h. The culture inoculum was adjusted to 0.5 McFarland (1.5 × 10^8^ CFU/mL) and was used to inoculate melted Muller Hinton agar at 50 °C. After medium solidification, wells were cut in agar using a cork borer. Wells were filled with 100 μL of the whole cell culture of *Lactobacillus* or cell-free supernatants (treated or untreated), and the plates were incubated aerobically at 37 °C for 24 h. Inhibition zones were measured to indicate the antimicrobial activity of the corresponding *Lactobacillus*. 

Stock solutions of treated or untreated *Lactobacillus* supernatants were maintained, and different concentrations 1/2, 1/4, 1/18, 1/16, and 1/32 were prepared. 

### 2.6. Effect of Lactobacillus Supernatant on Mature Biofilms

The mature biofilms of *P. mirabilis* isolates P2, P4, P14, P15, P23, P24, and P25 were formed in flat-bottomed 96-well microtiter plates. The overnight culture of each isolate was adjusted to 0.5 McFarland using Muller Hinton broth. Then, 100 µL of the diluted *Proteus* cultures was added to each well and incubated at 37 °C for 48 h. The planktonic cells were removed and the attached cells were gently washed twice with sterile physiological saline. Untreated or treated supernatants of *Lactobacillus* were then added to the wells and the plates were incubated at 37 °C for 24 h. The biofilm of each *Proteus* isolate without *Lactobacillus* supernatants was used as a positive control. The influence of different concentrations of *Lactobacillus* supernatants (treated or untreated) on the mature biofilm was detected using crystal violet microtiter plate assays. The treated and the mature *Proteus* biofilms were washed, fixed using methanol, and stained with crystal violet as described before [36].

The effects of different concentrations (1/2, 1/4, 1/18, 1/16, and 1/32) of treated and untreated supernatants of *L. casei* and *L. reuteri* on the mature biofilms of the *P. mirabilis* isolate P14 were also evaluated.

### 2.7. Anti-Adherence Effect of Lactobacillus Supernatants

In order to study the effect of *Lactobacillus* on biofilm formation, 100 µL of treated or untreated supernatants of *Lactobacillus* was mixed at low concentrations (1/8, 1/16 and 1/32) with 100 µL of the *P. mirabilis* isolate P14 (with inoculum size 1.5 × 10^8^ CFU/mL). The microtiter plate was incubated for 48 h at 37 °C and the biofilm formation was detected using the crystal violet microtiter plate method [17]. Wells containing the *Proteus* isolate P14 in contact with different concentrations of *Lactobacillus* supernatant (At) were compared to wells containing *Proteus* culture without the *Lactobacillus* supernatant (control, Ac). The percent reduction in biofilm formation was calculated as follows = ((Ac − At)/Ac) ×100.

### 2.8. Scanning Electron Microscopy (SEM)

The overnight culture of P14 was diluted to 0.5 McFarland using tryptic soy broth (TSB). Similarly, cultures of the tested *Lactobacillus* strains were diluted in MRS and co-cultured with equal volumes of P14 (1:1) in sterile six well plates (Greiner Bio-One, Kremsmünster, Austria) at 37 °C for 24 h. The control wells containing P14 only with MRS and TSB media were also prepared. A clean sterile cover slide was added to each well. The slides were removed and washed gently with phosphate buffered saline (PBS) to remove the planktonic cells. The biofilm was fixed and prepared for examination by SEM (JSM-7600F, JEOL USA, INC., Peabody, MA, USA) as previously described [37].

### 2.9. Statistical Analysis

Statistical analysis was performed using one-way ANOVA in order to compare the effect of *Lactobacillus*, cell-free, and treated supernatants on the mature biofilm and on the adhesion of *P. mirabilis* isolates. A *p* value of <0.05 indicated statistical significance. 

## 3. Results

### 3.1. Antimicrobial Susceptibility and Biofilm Formation of P. mirabilis isolates

Activity of the tested antimicrobial agents against *P. mirabilis* isolates was evaluated according to CLSI standards [32]. As shown in Table 1, 86% of the tested *P. mirabilis* isolates were resistant to amoxicillin-clavulanic, 57% were resistant to cefotaxime, 57% were resistant to ceftazidime, 71% were resistant to ciprofloxacin, 57% were resistant to amikacin, 43% were resistant to imipenem, and 0% were resistant to cefoxitin. Furthermore, isolate P14 was resistant to cefotaxime, ceftazidime, amoxicillin-clavulanic acid, ciprofloxacin, and amikacin and intermediately resistant to imipenem (Table 1). In contrast, isolate P2 was sensitive to all the assessed antimicrobials (Table 1).

*P. mirabilis* isolates P2, P4, P14, P24 and P25 showed strong biofilm formation. Isolates P15 and P23 showed moderate biofilm formation. Isolate P14 showed the strongest biofilm formation and was resistant to the tested antimicrobials. Isolate P2 showed a strong biofilm formation, but was sensitive to all assessed antimicrobials. 

### 3.2. Antimicrobial Activities of L. casei and L. reuteri against P. mirabilis Isolates

The untreated supernatants of *L. casei* and *L. reuteri* had inhibitory effects on the tested *P. mirabilis* isolates (Table 2). The treated (pH-adjusted) supernatants of *L. reuteri* were effective against almost all tested *P. mirabilis* isolates with inhibition zone diameters ranging from 12 to 16 mm. The treated (pH-adjusted) supernatants of *L. casei* was effective against four isolates (P2, P14, P15 and P24) with inhibition zone diameters ranging from 13 to 16 mm. Notably, the treated supernatants of *L. casei* and *L. reuteri* were effective against the multidrug resistant (MDR) *Proteus* isolate P14 with inhibition zone diameters ranging from 14 to 15 mm (Table 2).

### 3.3. Effect of Lactobacillus Supernatants on Mature Biofilm Formation of P. mirabilis Isolates

**Figure 1:**
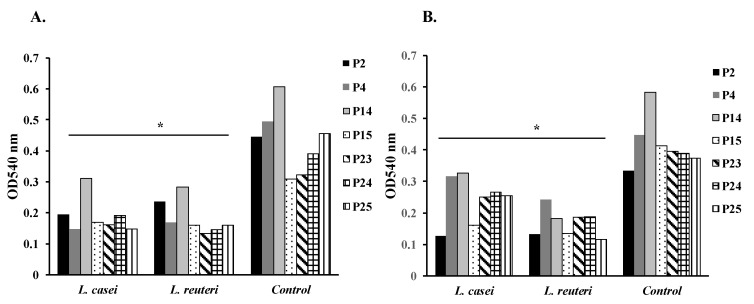
Effect of supernatants of *L. casei* and *L. reuteri* on mature biofilm formation of *Proteus mirabilis* isolates P2, P4, P14, P15, P23, P24, and P25 (* *p* < 0.05). (**A**) Effect of untreated supernatants of *L. casei* and *L. reuteri* on mature biofilm formation of *Proteus mirabilis* isolates P2, P4, P14, P15, P23, P24, and P25 (* *p* < 0.05). (**B**) Effect of treated supernatants of *L. casei* and *L. reuteri* on mature biofilm formation of *Proteus mirabilis* isolates P2, P4, P14, P15, P23, P24, and P25 (* *p* < 0.05).

The effect of treated and untreated supernatants of *L. casei* and *L. reuteri* on mature biofilm formation of *P. mirabilis* isolates P2, P4, P14, P15, P23, P24, and P25 was studied (Figure 1).

The untreated supernatants of *L. casei* significantly reduced biofilm formation of isolates P2, P4, P14, P15, P23, P24, and P25 by 56%, 70%, 48%, 45%, 49%, 50%, and 67%, respectively (*p* < 0.05) (Figure 1A). Furthermore, the treated supernatants of *L. casei* significantly reduced biofilm formation of isolates P2, P4, P14, P15, P23, P24, and P25 by 61%, 29%, 40%, 60%, 36%, 32%, and 32%, respectively (*p* < 0.05) (Figure 1B).

Similarly, the untreated supernatants of *L. reuteri* significantly reduced biofilm formation of isolates P2, P4, P14, P15, P23, P24, and P25 by 46%, 65%, 52%, 48%, 58%, 62%, and 65%, respectively (*p* < 0.05) (Figure 1A). Moreover, the treated supernatants of *L. reuteri* significantly reduced biofilm formation of isolates P2, P4, P14, P15, P23, P24, and P25 by 60%, 45%, 66%, 67%, 52%, 52%, and 69%, respectively (*p* < 0.05) (Figure 1B).

In brief, the treated and the untreated supernatants of *L. casei*, and *L. reuteri* caused significant reductions of mature biofilm formation in the tested *P. mirabilis* isolates (*p* < 0.05) (Figure 1).

**Figure 2:**
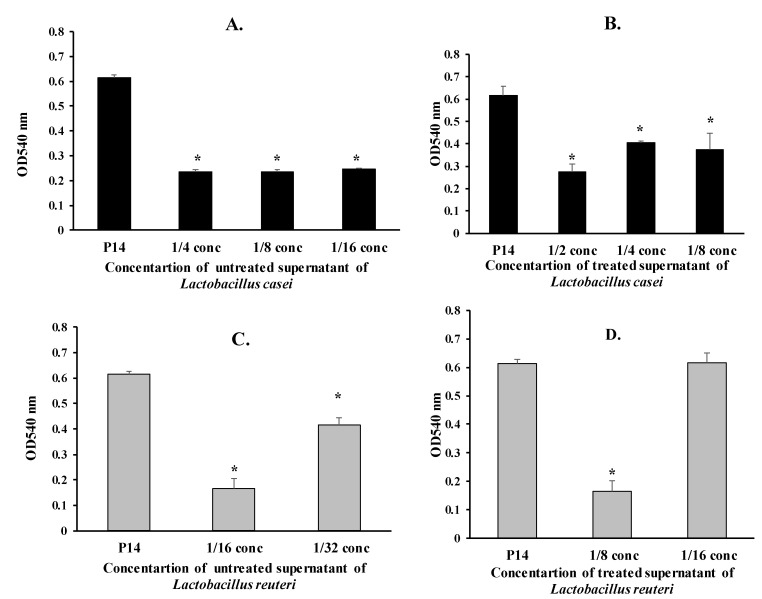
Effect of supernatants of *L. casei* and *L. reuteri* on mature biofilm formation of the *P. mirabilis* isolate P14 (* *p* < 0.05). (**A**) Effect of untreated supernatants of *L. casei* on mature biofilm formation of the *Proteus* isolate P14 (* *p* < 0.05). (**B**) Effect of treated supernatants of *L. casei* on mature biofilm formation of the *Proteus* isolate P14 (* *p* < 0.05). (**C**) Effect of untreated supernatants of *L. reuteri* on mature biofilm formation of the *Proteus* isolate P14 (* *p* < 0.05). (**D**) Effect of treated supernatants of *L. reuteri* on mature biofilm formation of the *Proteus* isolate P14 (* *p* < 0.05).

Next, we tested various concentrations of treated and untreated supernatants of *L. casei* and *L. reuteri* on mature biofilm formation of the MDR isolate P14. The diluted untreated supernatants (1/4, 1/8, and 1/16) of *L. casei* caused a significant reduction of biofilm formation in the P14 isolate by 61%, 61%, and 60%, respectively (*p* < 0.05) (Figure 2A). Similarly, the diluted treated supernatants (1/2, 1/4, 1/8) of *L. casei* significantly reduced biofilm formation in the P14 isolate by 55%, 33%, and 39%, respectively (*p* < 0.05) (Figure 2B.). 

The 1/16 and 1/32 concentrations of the untreated supernatants of *L. reuteri* caused a significant reduction in biofilm formation of the P14 isolate by 73% and 32%, respectively (*p* < 0.05) (Figure 2C). The 1/8 concentration of the treated supernatants of *L. reuteri* significantly reduced biofilm formation of the P14 isolate by 73% (*p* < 0.05) (Figure 2D). 

### 3.4. Anti-Adherence Effect of Lactobacillus Supernatants

**Figure 3:**
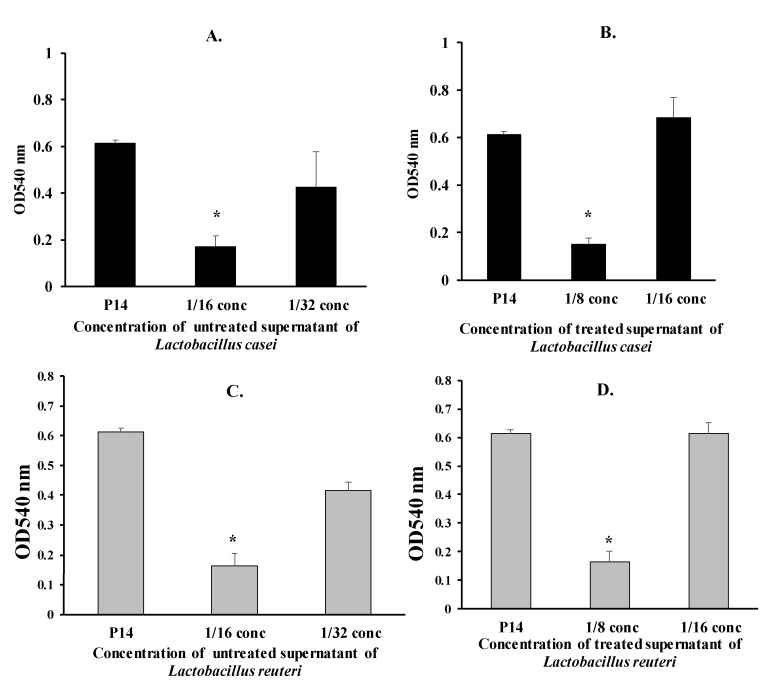
Adherence of the *Proteus* isolate P14 in the presence of supernatants of *L. casei* and *L. reuteri* (* *p* < 0.05). (**A**) Adherence of the *Proteus* isolate P14 in the presence of untreated supernatants of *L. casei* (* *p* < 0.05). (**B**) Adherence of the *Proteus* isolate P14 in the presence of treated supernatants of *L. casei* (* *p* < 0.05). (**C**) Adherence of the *Proteus* isolate P14 in the presence of untreated supernatants of *L. reuteri* (* *p* < 0.05). (**D**) Adherence of the *Proteus* isolate P14 in the presence of treated supernatants of *L. reuteri* (* *p* < 0.05).

The anti-adherence effect of diluted *Lactobacillus* supernatants (treated and untreated) on the P14 isolate was assessed. As in Figure 3, the untreated supernatants of *L. casei* and *L. reuteri* significantly reduced the adhesion of the P14 isolate (1/16 concentration) by 72% and 73%, respectively (*p* < 0.01) (Figure 3A,C). The anti-adherence effect of the untreated supernatants on the P14 isolate decreased by further diluting the supernatant. 

As in Figure 3, the treated supernatants of *L. casei* and *L. reuteri* significantly reduced biofilm formation of the P14 isolate (1/8 concentration) by 75% and 73%, respectively (*p* < 0.01) (Figure 3B,D). 

### 3.5. Scanning Electron Micrographs

**Figure 4:**
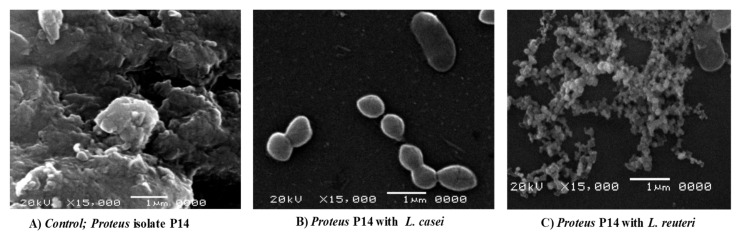
Scanning Electron micrographs (SEM) (magnification: × 15,000). (**A**) Biofilm formation of the P14 isolate in the absence of *Lactobacillus* spp. (control). (**B**) Biofilm formation of the P14 isolate in the presence of *L. casei*. (**C**) Biofilm formation of the P14 isolate in the presence of *L. reuteri*.

As in Figure 4A, there is a dense mass of biofilm-forming *P. mirabilis* P14 (as a positive control). Co-culture of *L. casei* with *P. mirabilis* P14 caused the complete elimination of biofilm formation by P14 (Figure 4B). Co-culture of *L. reuteri* with *P. mirabilis* P14 showed scattered cells and a loose biofilm architecture but no dense aggregates (Figure 4C). 

## 4. Discussion

Bacterial resistance is one of the major public health problems. It includes the emergence of MDR pathogens, such as resistant isolates of *Proteus mirabilis*, which have been associated with urinary tract infections and nosocomial infections worldwide [4,38]. *P. mirabilis* isolates have been reported to be resistant to penicillins [39]. They also show a high incidence of resistance to cephalosporins, carbapenems, and quinolones [4,40]. In the current study, a high percentage of the isolates was resistant or intermediately resistant to amoxicillin-clavulanic acid and cefotaxime. There was also significant resistance to ceftazidime, ciprofloxacin, and amikacin (Table 1). It is noteworthy that the isolate P14 was resistant to three different groups of antibiotics with various mechanisms of action (Table 1). Cefotaxime-resistant *P. mirabilis* has been previously described [41]. Kwiecińska-Piróg et al. reported that *P. mirabilis* showed resistance against ceftazidime and ciprofloxacin [42]. Amikacin resistance has been shown to be associated with extended spectrum beta-lactamase (ESBL(-producing isolates of *P. mirabilis*, which were resistant to amikacin (85.1%) [43].

Urinary tract infection with *P. mirabilis* is associated with biofilm formation and accumulation of the polysaccharide matrix [44]. This is followed by urease production, increase in the pH, attraction of calcium and magnesium ions, and development of crystals [44]. The deposition of crystals within the biofilm can cause catheter blockage and urinary retention. This is because *P. mirabilis* possesses various virulence factors (lipopolysaccharide, quorum sensing autoinducers, pili, adhesin, and other proteins) that enhance adhesion and crystalline biofilm formation on the abiotic surfaces of urinary catheters [45]. It has been reported that 48% of the isolated proteus species were biofilm producers [42]. The *Proteus*-biofilm assembly is dangerous, as it interferes with microbial penetration, increases antimicrobial resistance, and renders therapeutic treatments ineffective, which encourages the development and chronicity of infections [46]. 

Moreover, pathogenicity of *P. mirabilis* is enhanced by the complex architecture of its biofilm, which is characterized by a high ability for adapting to different environmental conditions, biocides, and antimicrobials [47,48]. Isolates in this study were able to form strong or moderate biofilms. This was true even for the antibiotic-sensitive isolate P2, which was observed to have a strong biofilm-forming ability (Table 1). Typically, the biofilm-producing isolates are more resistant to antibiotics than the non-biofilm producing isolates [49]. The reason is that the development of complex biofilm structures can convey protection to the internal cells from antimicrobials [50]. It can also support the persistence of *P. mirabilis* in the host cells [50].

Given the above, alternative treatments are required for the management of *P. mirabilis* infections and the disruption of its biofilm architecture. Probiotics as *Lactobacilli*, have been used for the treatment of burn infections and have been shown to interfere with activity of *Pseudomonas aeruginosa* [51,52]. *Lactobacilli* have also been used for the management of recurrent urinary tract infections caused by *E. coli* [53]. The antimicrobial activity of *L. casei* and *L. reuteri* against various enteropathogenic infections have been previously reported [10]. Their antimicrobial activities against uropathogens have also been reported [20]. *L. reuteri* has been shown to be effective against *Escherichia coli* and *Listeria monocytogenes* [54]. Thus, it was important to examine the antimicrobial and antibiofilm activities of *Lactobacillus* on *P. mirabilis* clinical isolates. We examined the cultures and the cell-free supernatants of *L. casei*, and *L. reuteri* against *P. mirabilis* isolates. 

In our study, the treated supernatants of *L. casei* and *L. reuteri* retained antimicrobial activities against the sensitive P2 isolate and the other resistant *Proteus mirabilis* isolates (Table 2). Notably, treated supernatants of *L. casei* and *L. reuteri* retained significant antibiofilm and anti-adherence activities against P14 at 1/8 concentration (Figure 2 and Figure 3). In addition, *L. casei* and *L. reuteri* had significant inhibitory effects on the typical biofilm formed by the MDR isolate P14, as detected by SEM (Figure 4). In another study, the untreated supernatants of fecal *Lactobacilli* caused an 85 to 95% reduction in biofilm formation of *Vibrio cholerae* isolates [27]. Similarly, the pH-adjusted supernatants of fecal Lactobacilli significantly reduced biofilm formation of Vibrio cholerae by 50–75% [27]. Additionally, Koohestani and colleagues showed that the untreated supernatants of *L. casei* significantly reduced biofilm formation of *S. aureus* [55]. Probiotic lactobacilli have been shown to negatively impact growth and biofilm formation of *Streptococcus mutans* [34], pathogens in the oral cavity [56], and *Salmonella enterica* serovar *Typhimurium* [28]. Moreover, probiotic lactobacilli inhibited cancer cells of the human colonic carcinoma cell line HT-29 [23]. Lastly, *Lactobacillus reuteri* DPC16 supernatants have been shown to exhibit activity against *Escherichia coli*, *S. aureus*, *Salmonella derby*, and *Listeria monocytogenes*. This is consistent with results in our study in which the untreated supernatants of *L. casei* reduced biofilm formation of *P. mirabilis* isolates by 45–67%.

It is important to mention that the use of probiotic *Lactobacillus* cell cultures and supernatants was proposed as an alternative to traditional antibiotic therapy against *P. mirabilis*. In other words, it was proposed as a way to avoid the adverse effects of antibiotic therapy and to circumvent the impact of antibiotic resistance developed by the microbe. Indeed, we saw a significant inhibitory effect of *Lactobacillus* cell cultures and supernatants on *P. mirabilis* biofilm formation and adherence. Future studies could examine the effect of *Lactobacillus* supernatants on the susceptibility of *P. mirabilis* to the tested antimicrobials. In this case, the focus would be on the possible synergistic effects of combining probiotic *Lactobacillus* supernatants with anti-*Proteus* agents. 

The antimicrobial, antibiofilm, and anti-adherence activities of probiotic *L. casei* and *L. reuteri* used in this study could be attributed to their secreted biosurfactants [15], S-layer proteins [57,58], surface-acting proteins (such as enolase) [59], and peptidoglycan-binding proteins [60]. 

## 5. Conclusions

The pathogenicity and virulence of *Proteus* infections includes a biofilm-forming ability that enables serious urinary tract infection. Isolates in this study showed multidrug resistance (MDR) against more than one antimicrobial agent, highlighting the importance of the development of alternative and adjuvant treatments for the efficient management of *P. mirabilis* infections. Using cell cultures, cell-free supernatants, and pH-adjusted supernatants, we have shown that *L. casei* DSM 20011 and *L. reuteri* DSM 20016 exhibit antimicrobial, anti-adherence, and antibiofilm activities against MDR *P. mirabilis*. Thus, *L. casei* and *L. reuteri* could be utilized to combat *Proteus*-associated urinary tract infections. 

## Figures and Tables

**Table 1:** Antimicrobial susceptibility patterns of *P. mirabilis* isolates.

Isolate No.	FOX 30 mg	CAZ 30 mg	CTX 30 mg	AMC 30 mg	IMP 10 mg	CIP 5 mg	AK 30 mg	Biofilm Formation
**P2**	S	S	S	S	S	S	S	Strong
**P4**	S	S	S	R	I	I	S	Strong
**P14**	S	R	R	R	I	R	R	Strong
**P15**	S	S	S	I	I	S	S	Moderate
**P23**	S	R	R	R	S	R	R	Moderate
**P24**	S	R	R	R	S	R	R	Strong
**P25**	S	R	R	R	S	R	R	Strong

FOX: Cefoxitin; CAZ: Ceftazidime; CTX: Cefotaxime; AMC: Amoxicillin-Clavulanic acid; IMP: Imipenem; CIP: Ciprofloxacin and AK: Amikacin. S: Sensitive, R: Resistant, and I: Intermediate.

**Table 2:** Antimicrobial activities of *L. casei* and *L. reuteri* against *P. mirabilis* isolates.

Inhibition Zone Diameter (mm)						
Isolate No.	*L. casei* DSM 20011			*L. reuteri* DSM 20016		
	C	T	U	C	T	U
**P2**	20	13	20	20	15	20
**P4**	20	-	21	21	16	20
**P14**	18	14	18	20	14	20
**P15**	20	16	20	20	15	18
**P23**	19	-	20	20	15	20
**P24**	20	14	20	20	12	19
**P25**	20	-	20	20	-	18

C: The *Lactobacillus* Culture; T: The treated (pH-adjusted) cell-free supernatants of *Lactobacillus*; U: The untreated cell-free supernatants of *Lactobacillus*; -: No effect.
